# Clinical and Financial Value of Cardiac Point-of-Care Ultrasound During Traumatic Cardiac Arrest

**DOI:** 10.5811/westjem.61896

**Published:** 2026-09-10

**Authors:** Donias Doko, Zachary Boivin, Kevin M. Duignan, Nishant Merchant, Trent She

**Affiliations:** * University of Connecticut, Emergency Medicine Residency Program, Farmington, Connecticut; † Yale School of Medicine, Department of Emergency Medicine, New Haven, Connecticut; ‡ Hartford Hospital, Department of Acute Care Surgery, Hartford, Connecticut; § Hartford Hospital, Department of Emergency Medicine, Hartford, Connecticut

## Abstract

**Introduction:**

Traumatic cardiac arrest is associated with poor survival and significant consumption of hospital resources. Determining which patients have survivable injuries allows for judicious use of resources. Our aim was to determine whether cardiac activity on point-of-care ultrasound (POCUS) during traumatic cardiac arrest was associated with increased patient survival and the charges of continued resuscitation in patients that do have cardiac activity on POCUS.

**Methods:**

We conducted this single-center retrospective study at an urban, Level I trauma center of consecutive patients arriving in traumatic cardiac arrest from August 2016–December 2023 who had a cardiac POCUS performed during the initial resuscitation efforts. Our primary outcome was to determine whether cardiac activity on POCUS during the arrest was associated with increased patient survival to neurologically intact hospital discharge. Secondary objectives included determining whether there was a correlation between cardiac activity on POCUS during traumatic cardiac arrest and survival to hospital admission. Additionally, we evaluated hospital charges and length of stay associated with continued resuscitation in patients who demonstrated cardiac activity on ultrasound.

**Results:**

We identified 134 patients in traumatic cardiac arrest, of whom 74 (55.2%) had a cardiac POCUS performed and met inclusion criteria. A total of 17 patients had cardiac activity noted on the initial POCUS, with two (11%) surviving to hospital discharge (95% CI, 1.3–39.7). The two survivors initially categorized as being in traumatic cardiac arrest were ultimately found to have medical etiologies and not trauma. Of the 57 patients who had no cardiac activity noted on POCUS, none survived to hospital discharge (95% CI, 0–6.3%). More patients survived to hospital admission who had cardiac activity on their initial POCUS compared with those who had no cardiac activity (odds ratio [OR], 49.8; 95% CI, 5.5–446.9; P < .001). More patients survived to hospital discharge as well, but this was not statistically significant (OR, 18.6; 95% CI, 0.84–406.7, P = .06). Traumatic cardiac arrest patients who had present cardiac activity had longer code times prior to terminating efforts (26.6 vs 7.7 minutes, P < .001) and had higher overall charges per patient ($91,321 vs $10,032), P < .001).

**Conclusion:**

The absence of cardiac activity on POCUS in patients with traumatic cardiac activity is likely to be fatal. The presence of cardiac activity was associated with increased resuscitation times, charges, and survival to hospital admission but not necessarily to hospital discharge.

## INTRODUCTION

Traumatic cardiac arrest is one of the highest acuity conditions faced by both surgery and emergency physicians. Although both Advanced Cardiac Life Support (ACLS) and Advanced Trauma Life Support (ATLS) guidelines exist to aid management, outcomes are frequently poor, with only 5–12% of patients surviving to hospital discharge.^1^,^2^ Significant resource use, both in terms of physicians’ time and hospital consumables, occurs during these resuscitations and can lead to downstream issues with future patients.^3^ It is therefore important that physicians have a way to differentiate which patients presenting in traumatic cardiac arrest have potentially survivable injuries and which do not.

Point-of-care ultrasound (POCUS) can rapidly assess multiple organ systems for traumatic injury at bedside. In nontraumatic cardiac arrest, POCUS is frequently used and accepted as a key clinical tool. Up to 91% of emergency physicians use ultrasound to assist in terminating resuscitation efforts, and presence of cardiac activity has a pooled odds ratio of 12.4 for return of spontaneous circulation (ROSC)_._^4^,^5^ A 793-patient prospective study in nontraumatic cardiac arrest found that cardiac activity during initial resuscitation efforts was associated with increased survival to hospital admission (odds ratio [OR], 3.6; 95% CI, 2.2–5.9) and hospital discharge (OR, 5.7; 95% CI, 1.5–21.9) whereas only 0.6% (95% CI, 0.3–2.3) of patients with absent cardiac activity on initial POCUS survived to hospital discharge.^6^–^7^ However, the optimal application of cardiac POCUS in traumatic cardiac arrest is less clear. There is some evidence suggesting that the absence of cardiac activity on POCUS is associated with nonsurvivable injury, with sensitivities ranging from 73%–91% and specificities ranging from 91–98% but with relatively heterogenous populations.^8^–^13^

### Outcomes

Our primary outcome was to determine whether cardiac activity on POCUS during traumatic cardiac arrest was associated with increased patient survival to neurologically intact hospital discharge. Secondary objectives included determining whether there was an association between cardiac activity on POCUS during traumatic cardiac arrest and survival to hospital admission, whether the absence of cardiac activity was associated with termination of resuscitative efforts, and the relative hospital-visit charges, both material and time-based, of continued resuscitation efforts in patients who do have cardiac activity on POCUS. Resuscitative procedures assessed included the following: airway; chest tube; central venous access; blood administration; and emergency department (ED) thoracotomies. We also compared hospital length of stay (LOS) in days for patients who were admitted. Our a priori hypothesis was that cardiac activity on POCUS during traumatic cardiac arrest would be associated with increased patient survival to hospital admission and neurologically intact discharge but at an increased charge.

**Population Health Research Capsule**

What do we already know about this issue?

*Traumatic cardiac arrest has poor survival and high resource use. Cardiac point-of-care ultrasound (POCUS) may identify patients with survivable injury during resuscitation*.

What was the research question?

*Is cardiac activity on POCUS associated with survival and charges in traumatic cardiac arrest?*

What was the major finding of the study?

*Cardiac activity on POCUS was associated with survival to hospital admission: 47% vs 1.8%; 95% CI, 5.5–446.9; P < .001*.

How does this improve population health?

*Early cardiac POCUS may guide traumatic cardiac arrest resuscitation, limiting futile care and conserving emergency department/trauma resources without missed survival*.

## METHODS

### Adherence to Methodologic Standards

The following optimal methodological standards were adhered to in this study.^14^ All abstractors (DD, ZB, KD) were trained via a one-hour instructional session on data collection in the hospital’s electronic health record (EHR) prior to collecting any data. The inclusion and exclusion criteria are explicitly defined below in the section on inclusion and exclusion criteria. Relevant variables are defined in the sections below. Data abstractors used a common data abstraction form. A separate investigator (TS) who was not part of the initial chart review then re-reviewed charts to ensure consistency and also reviewed overall data collection at regular intervals. All identified cardiac POCUS images were reviewed by an ultrasound fellowship-trained attending with experience performing and interpreting more than 1,000 cardiac POCUS examinations. Institutional review board approval at our local institution was obtained. Any missing data were noted, discussed with the abstractor and re-reviewer, and resolved by consensus.

### Setting

This was a single-center, retrospective study at an academic, tertiary care, urban Level I trauma center, with greater than 110,000 ED visits and approximately 20–25 traumatic cardiac arrest cases in approximately 550 full trauma responses a year. Point-of-care ultrasound examinations are typically performed by an emergency medicine (EM) intern who has had a dedicated one-hour training session on cardiac POCUS with both a lecture and hands-on component. These exams are supervised by an EM fellow or attending. All ED attendings and fellows are globally credentialed to perform POCUS via the American Board of Emergency Medicine and receive training in POCUS as a condition for their board certification. Emergency department residents participate in 16 hours of dedicated ultrasound training during their first month and at least four weeks of dedicated POCUS training by the end of their second year, and they must perform a minimum of 500 POCUS examinations during their residency as a requirement for graduation. Occasionally, POCUS examinations are performed by a member of the trauma team: either a nurse practitioner, physician assistant, rotating EM or surgical resident, or the attending trauma surgeon.

Our ED has an active POCUS program with approximately 9,000 POCUS examinations performed annually, as well as an accredited three-year EM residency and a one-year emergency ultrasound fellowship. Point-of-care ultrasound was conducted with either a Sonosite Xporte (FUJIFILM Sonosite, Bothell, WA) or a Philips Sparq (Philips, Andover, MA). Quality assurance was performed by clinical ultrasound fellowship-trained faculty members. Cardiac POCUS was performed typically within the first 2–4 minutes during the first or second pulse check in all traumatic cardiac arrest resuscitation efforts. Subsequent POCUS, including the remaining views of the extended focused assessment for sonography in trauma (eFAST) exams, may have been obtained during subsequent pulse checks.

### Inclusion and Exclusion Criteria

Our research team conducted a retrospective chart review to identify all traumatic cardiac arrest patients presenting to our ED from August 1, 2016–December 31, 2023. Patients were identified consecutively using *International Classification of Diseases, 10^th.^ Revision*, (*ICD-10*) codes for cardiac arrest. Inclusion criteria were all patients at least 18 years of age who presented in traumatic cardiac arrest or who went into traumatic cardiac arrest in the ED who also had a cardiac POCUS conducted during the resuscitation. We accepted dedicated cardiac POCUS clips as well as cardiac POCUS clips that were in the context of an eFAST exam, provided that it was the first POCUS exam in the resuscitation. We defined cardiac arrest as a patient who had an absent palpable pulse and a cardiac rhythm requiring cardiopulmonary resuscitation or ACLS. The cardiac arrest was deemed to be traumatic if there was an external cause, blunt and penetrating, to the patient’s presenting symptoms (such as a gunshot wound or motor vehicle collision). In some cases, determining the exact cause of cardiac arrest at the time of ED presentation was difficult; in these cases, we elected to include patients if the trauma surgery team was called to assist in resuscitation efforts based on our hospital’s trauma activation guidelines (Table 1).

Patients were excluded if they had any of the following conditions: ROSC prior to ED arrival; a cardiac arrest after hospital admission; an advanced directive invoked during resuscitation. efforts; a nontraumatic cause for cardiac arrest (based on chart review); or lack of trauma team involvement in the patient’s care.

A primary goal of POCUS in cardiac arrest is to assess for organized cardiac activity, which we defined as movement of the heart with sufficient cardiac output to sustain life, as defined in previous literature.^15^ However, because there is no standard for the precise sonographic findings that constitute organized cardiac activity, we left this determination to the clinicians for each patient at the time of resuscitation. efforts, determined via physician documentation.^16^ As there was no standardized POCUS workflow for much of the study period (our ED only obtained a dedicated POCUS workflow in October 2021), many cardiac POCUS images were lost to review by study investigators. Nevertheless, study investigators did attempt to locate all cardiac POCUS images associated with patients in this study by review of our institution’s picture archiving and communication system

Survival to hospital admission was defined as having all three of the following: ROSC; admission orders placed; and transfer out of the ED. We included patients who went directly to the operating room in the category of those who survived to hospital admission. Survival to hospital discharge was defined as a patient who survived to hospital admission and was then discharged to a definitive location (eg, rehab facility, home). We defined neurologically intact hospital discharge as a patient who met the criteria for survival to hospital discharge and who also had a Cerebral Performance Category score ≤ 2, as calculated by a member of the research team.^17^ We evaluated resource utilization procedurally and financially by tracking the number of advanced airway procedures, central venous access placements, chest tube insertions, and thoracotomies performed, as well as total charges per hospital visit.

### Chart Review

After a joint discussion with all study members and a one-hour training session, study investigators manually reviewed the EHR within the study period to identify patients who had an *ICD-10* code consistent with cardiac arrest. A separate investigator who was not part of the initial chart review then re-reviewed approximately 10% of the charts to ensure consistency, and any errors discovered were corrected by re-review and consensus between the initial abstractors and the re-reviewer. The Cohen kappa (κ) was 0.89 between the initial reviewer and subsequent re-reviewer for all charted variables.

The study investigators reviewed each case of traumatic cardiac arrest as compiled in an existing database of the hospital’s EHR based on *ICD-10* codes for cardiac arrest, which is kept for quality assurance purposes. Data was obtained from the initial patient encounter, patient records, emergency medical services records, and hospital billing data. Our team reviewed charts for demographics, comorbidities, medications administered, procedures performed, POCUS findings, and patient outcomes. Additional data such as comorbidities was obtained by chart review to obtain data points not available during the initial patient resuscitation.

### Statistical Analysis

Outcome data was reported as either discrete or continuous numerical data and were stored in Microsoft Excel 2017 (Microsoft Corporation, Redmond, WA). Tests of normality were conducted on all outcome data using the Shapiro-Wilk test. We subsequently analyzed normally distributed data with a two-tailed Student *t*-test, while nonnormally distributed data were analyzed with a Mann-Whitney *U* test. All analyses were conducted using SPSS version 28 (IBM Corporation, Armonk, NY). A *P* value < .05 was considered statistically significant.

## RESULTS

A total of 134 patients with traumatic cardiac arrest were identified during the study period, of whom 79 (59.3%) had cardiac POCUS performed. Two patients had ROSC immediately upon ED arrival and were excluded. Three patients were excluded for incomplete ultrasound documentation, leaving a total of 74 patients in the final study analysis (Figure). The characteristics of the included and excluded patients are listed in Table 2.

The average age of included patients was 48.1 (standard deviation [SD] 22.0), 58 (73.4%) were male, 15 (20.3%) had penetrating trauma, and 59 (79.8%) blunt trauma.

A total of 17 patients (23.0%) had cardiac activity noted on the initial POCUS with 8 surviving to hospital admission (47% of group, 10.8% of study population) and 2 surviving to discharge (11.8% of group, 2.7% of study population, 95% CI, 0.24–7.22). Of the 57 patients (77.0%) who had no cardiac activity noted on POCUS, only one survived to hospital admission (1.8% of group, 1.4% of study population), and none survived to discharge (95% CI, 0–6.3%). Compared with those patients who had cardiac activity on their initial POCUS, those with no cardiac activity noted had higher rates of survival to hospital admission (47% compared with 1.8% absolute risk reduction [ARR]) and to hospital discharge [11.8% compared with 0% ARR]). The difference in survival to hospital admission was statistically significant (odds ratio [OR], 49.78; 95% CI, 5.54–446.92], *P* < .001), and the difference in survival to hospital discharge was ultimately not statistically significant (OR, 18.55; 95% CI, 0.84–406.7, *P* = .06). Of the patients with cardiac activity who survived to hospital admission, LOS on average was 8.37 days (SD 9.71). The single patient without cardiac activity on POCUS who survived to hospital admission died one day later.

Of the 74 included patients with a cardiac POCUS, only 13 (17.5%) had saved clips available to the study team for review. On re-review of these images, 8 of the 13 patients had no cardiac activity documented by the clinical team, and the other 5 had cardiac activity documented. On image review, three of the eight patients who were documented to have absent cardiac activity actually had cardiac activity; all five patients who had cardiac activity did indeed have cardiac activity on review (κ = 0.56). In addition, two of the patients who had no cardiac activity were found to have a pericardial effusion. One patient had a small pericardial effusion (maximum diastolic diameter < 1.0 cm), and one patient had a moderate pericardial effusion (maximum diastolic diameter 1.0–2.0). Both patients presented with traumatic cardiac arrest secondary to penetrating trauma. Investigators could not assess for cardiac tamponade because complete cardiac standstill precluded evaluation for right ventricular collapse on ultrasound. Despite variability of POCUS findings on re-review, we opted to keep patients in their initial groups based on what the clinical team believed their POCUS findings to be during the initial resuscitation efforts.

There was a significant difference in the length of resuscitation efforts between patients with cardiac activity on POCUS (26.59 minutes) compared with those without cardiac activity on POCUS (7.67 minutes, *P* < .001). There was also a significant difference in charges, with patients with cardiac activity having an average total hospital charge of $91,321 (SD $133,402) per patient as compared to the patients without cardiac activity ($10,032 [$12,976]). Patients with cardiac activity had more blood products used on initial resuscitative effort, with 0.94 units of packed red blood cells (pRBC) compared with 0.44 pRBCs per patient without cardiac activity (*P* = .01). Patients with cardiac activity also underwent more total procedures, including central venous catheterizations and surgical interventions in the operating room (Table 3).

Additional analysis of hospital charges was conducted on the patients who died in the ED and those who survived to hospital admission (Table 4). Of the eight patients who survived to hospital admission with cardiac activity, the average charge of their total hospital stay was $180,991. The one person who survived to hospital admission with no cardiac activity had a total hospital stay charge of $69,805 (*P* < .001). The two patients who survived to hospital discharge had an average hospital charge of $346,329. Of the nine patients who died in the ED with cardiac activity on ultrasound, their hospital charge was significantly higher than the 56 patients who died in the ED with no cardiac activity ($27,024 vs $8,945, *P* < .001).

## DISCUSSION

### Interpretation and Comparison to Previous Studies

Despite ongoing medical improvements and advancements in ATLS care in both prehospital and in-hospital settings, patients with traumatic cardiac arrest still have a high mortality rate. Determining which may benefit from continued resuscitation efforts is a difficult decision dependent on multiple factors. Our data suggest that POCUS may assist in this decision, as the presence or absence of organized cardiac activity on POCUS may serve as a prognostic sign of successful resuscitation. For traumatic cardiac arrest, poor outcomes have been associated with cardiac standstill in previous research, including a 0% survival rate in a single-center study of 187 traumatic cardiac arrest patients with standstill. Cardiac motion predicting survival to hospital admission was 86% sensitive and 91% specific in another single-center study of 162 traumatic cardiac arrest patients. A meta-analysis of 710 patients found negligible rates of survival to hospital discharge when cardiac standstill was present. Our study mirrors these findings, demonstrating dismal survival to hospital admission among patients presenting with cardiac standstill.

We report on the charges associated with the presence or absence of cardiac activity, which was documented less commonly in the literature. We noted one prior study of 37 traumatic cardiac arrest patients who saw decreased hospital costs with performance of POCUS in traumatic cardiac arrest ($1,871 in the POCUS group compared with $3,040 in the non-POCUS group); however, the study did not consider the findings on POCUS but rather only the actual performance of ultrasound. We found no suitable benchmark for hospital charges during traumatic cardiac arrest with which to compare our findings.

### Summary of Main Findings and Strengths

The 57 patients without cardiac activity on POCUS all died regardless of rhythm or resuscitative effort, consistent with previous studies. This finding was ultimately not statistically significant (*P* = .06). Although the absence of cardiac activity had a high odds ratio (18.55) for nonsurvivability, there was also a wide confidence interval associated with this analysis (95% CI, 0.84–406.7).^24^ We therefore advocate for early performance of cardiac POCUS to determine the cardiac function of traumatic cardiac arrest patients. By doing so, significant resuscitative charges can be saved once the absence of cardiac activity is determined. By identifying the absence of cardiac activity on POCUS, approximately $18,000 in-hospital visit charges were saved per traumatic cardiac arrest patient, with likely no change in meaningful patient outcome.

One patient survived to hospital admission from this cohort—a 48-year-old female with a self-inflicted laceration to the left wrist who had significant anoxic brain injury as a sequela to injury. The family requested that no aggressive measures be taken in her care, and she died one day after hospital admission. Based on our findings, we suggest that if no cardiac movement is noted on POCUS during the initial traumatic cardiac arrest evaluation, further resuscitative efforts would likely be futile. Consideration should be given to terminating resuscitative efforts if no obvious reversible causes, such as pericardial tamponade, are noted on the primary and secondary survey.

We also found that the presence of cardiac activity on POCUS did not lead to a statistical increase in survival to hospital discharge (although a statistically significant increase in survival to hospital admission was noted). It is interesting to consider why traumatic cardiac arrest patients with cardiac activity on POCUS still have such poor outcomes. One possibility is that severe traumatic injuries carry a dismal prognosis and would have had poor outcomes regardless of the presence of cardiac activity on initial POCUS. Another possibility may be the lack of a formal definition of “cardiac activity.” While we relied on the clinical team for the interpretation of the cardiac POCUS, there is a wide range of cardiac movement that can be considered cardiac activity.^16^ For patients who survived to hospital admission, it remains unclear whether a specific acute event led to death or if it resulted from the inevitable progression of their pathophysiology.

### Clinical Implications

By using POCUS and terminating resuscitative efforts in traumatic cardiac arrest patients without cardiac activity, significant resources may be saved by the ED and the trauma team. Compared to those without cardiac activity, traumatic cardiac arrest patients with present cardiac activity experienced longer code times prior to terminating efforts (7.67 compared with 26.59 minutes, *P* < .001), had more units of red blood cells administered per patient (0.44 compared with 0.94, *P* < .001), and had more procedures performed (*P* = .01). Patients without cardiac activity incurred significantly lower charges than those with cardiac activity, both overall ($8,945 compared with $27,024; *P* < .001) and among those who died in the ED ($7,262 compared with $17,156; *P* < .001). We do not advocate for charge-sensitive care as a primary motivating factor for traumatic cardiac arrest resuscitation decisions, because these additional charges incurred for patients who had cardiac activity ultimately led to increased survivability.

### Research Implications

Future prospective trials with larger samples sizes are required to validate these findings. Standardizing the definition of cardiac activity and ensuring the storage of ultrasound clips for retrospective review will improve data reproducibility. Furthermore, the study only evaluated cardiac POCUS findings and did not report data from other ultrasound views. Although the identification of a pneumothorax or hemoperitoneum may alter resuscitative decisions, the presence or absence of cardiac activity remains the most critical decision point during traumatic cardiac arrest resuscitation efforts.

## LIMITATIONS

We excluded a substantial cohort of patients with traumatic cardiac arrest because POCUS was not performed. This excluded population likely represented a less severely injured cohort, as evidenced by their significantly higher rates of survival to hospital admission and discharge (Table 1). In these cases, clinicians may have prioritized immediate definitive management over diagnostic bedside imaging. Neither did we review the actual ultrasound images; instead, we relied upon the resuscitation team’s report and charting. Although POCUS is routinely used to assess cardiac function early in a resuscitation effort, the exact timing of the ultrasound evaluation was not recorded. Therefore, we could not determine whether POCUS findings served as the primary driver for continuing or terminating resuscitation efforts. Other components of the patient’s presentation undoubtedly affected resuscitation decision-making, which would also have impacted the financial charges each patient accumulated. However, early POCUS use during cardiac arrest is standard protocol at our institution.

Only 13 patients (17.6%) of the 74 in the cohort had saved images available for retrospective analysis. On image review, three of the eight patients documented as having absent cardiac activity demonstrated subtle yet definitive cardiac motion. It is unclear whether this subtle cardiac activity that was initially missed by clinicians would have resulted in a meaningful change in resuscitation but suggests that ultrasound interpretation was not perfect by the treating physicians. Extrapolating to our entire population, there likely were more cases of misinterpreted POCUS that may have impacted resuscitative decision-making. However, since decision-making is not solely reliant on POCUS in cases of traumatic cardiac arrest, the clinical outcomes may have been similar even if physicians had been aware of the correct POCUS interpretation. Nevertheless, this is a notable limitation of our study.

Pericardial effusions and cardiac tamponade can also be diagnosed with cardiac POCUS and represent a reversible cause of traumatic cardiac arrest that can be acted upon by the medical team. No clinically significant pericardial effusions were noted on chart review; however, upon review of available images by investigators, two pericardial effusions were identified. Both were in the setting of penetrating trauma_—_one small and one moderate_—_and in both cases, they were accompanied by cardiac standstill. Because both patients were in standstill, it is difficult to ascertain whether cardiac tamponade was the direct cause of mortality in either case. However, it is likely that more pericardial effusions may have been present in our study cohort, which is another notable limitation.

The classification of “traumatic” cardiac arrest can be difficult because preceding medical events may lead to subsequent traumatic events, and assessing which event is the larger contributor to cardiac arrest is often not possible. We elected to include all cardiac arrests with trauma team activation, even if no traumatic injuries were subsequently noted, because there is a detailed set of guidelines dictating such activations, including the ability for the initial clinical team to request or cancel a trauma team activation. Only two patients in our study cohort survived to hospital discharge. The first was a 55-year-old man in a low-speed motor vehicle collision. After he was resuscitated in the ED, he was found to have rib fractures and small apical pneumothoraxes thought to be secondary to chest compressions rather than an initial traumatic injury. During his hospitalization, the patient admitted to recreational polysubstance use, which the clinician then attributed to his cardiac arrest. He was discharged to a rehabilitation facility with a Cerebral Performance Category (CPC) score of 2. The second patient who survived to hospital discharge was a 54-year-old man who fell off a ladder and was found to be in cardiac arrest. He received bilateral needle decompressions in the field. In the ED, ROSC was achieved, and subsequent electrocardiogram revealed an ST-elevation myocardial infarction and a 100% occlusion in his circumflex artery. He was eventually discharged home with a CPC score of 1.

We suspect that the cardiac arrest in both patients was unlikely secondary to a traumatic cause. Because the trauma team was called to aid in the initial resuscitation and because the lack of initial traumatic injuries was not known until after ROSC was obtained, we chose to include these two patients in our cohort. Conversely, our research team did not review each individual case of nontraumatic cardiac arrest to determine whether there may have been a traumatic cause of injury. Therefore, it is likely that some of our nontraumatic cardiac arrests likely were indeed traumatic in nature, a determination that could not have been made by chart review alone.

Additionally, our study focused on a single center; other hospitals may have different trauma activation criteria leading to a potentially higher catchment for traumatic cardiac arrest. Because our trauma center does not mandate cardiac POCUS in every cardiac arrest, a significant number of traumatic cardiac arrest patients did not receive cardiac ultrasound. It is unclear why. Perhaps it may have been clinician comfort, or there was an identified nonsurvivable injury or other unfavorable characteristics of the resuscitation effort that led to termination, or even that ROSC was achieved prior to the ultrasound. Knowing the reasoning behind the lack of POCUS in our traumatic cardiac arrest populations would have helped strengthen our research. Because our chart reviewers were also authors, they were not blinded to the original study hypotheses, which may have introduced bias.

Our POCUS workflow during the study timeframe was an order-based system; consequently, we were able to determine only the physician who ordered the POCUS and not the one who actually performed the exam. Although the ED intern typically is assigned to perform the POCUS as part of their resuscitation duties, we were unable to verify whether the intern in fact performed the exam. In the setting of traumatic cardiac arrest, a more senior resident or attending will often dictate management of ultrasound. In addition, our nurse practitioners and physician assistants, as well as the trauma service clinicians, are not subject to the same formal credentialing guidelines as our ED residents, fellows, and attendings, which means they may have had varying levels of training if they were the ones who performed or supervised the POCUS.

We report medical charge data, which may approximate cost but does not represent true cost. Actual cost data are more difficult to obtain because of bundled payment structures and variation in reimbursement across patients and insurance plans. In addition, there was opportunity cost from the trauma team, which was not accounted for in our charge data.

It would be interesting in future research to determine differences between study groups in critical care or intensive care unit (ICU) LOS. Unfortunately, our dataset does not readily allow us to distinguish ICU LOS from stepdown or floor LOS, because our EHR does not provide a reliable way to determine exactly when patients were formally transferred from the ICU to stepdown or floor status. Perhaps, this topic can be more formally addressed in the future

Some of our conclusions are clearly limited by a low sample size, given the single-center nature of our study. A recent meta-analysis of trauma centers that had patients with traumatic cardiac arrest showed varying patient volumes, including 88 patients over a three-year period in three hospitals in France; 588 patients in a six-year period in a single hospital in New Orleans, LA; 463 patients in a four-year period in five hospitals in Taiwan and 284 patients in a 10-year study period in a single hospital in Sweden.^25^–^29^ We had 134 patients with traumatic cardiac arrest over an approximate 6.5-year study timeframe, which represented a slightly lower annual average per hospital. However, as previously noted, the number of traumatic cardiac arrests per trauma center can vary significantly. In addition, the actual frequency of use of cardiac POCUS in these patients can also vary significantly. Although literature exists on the use of cardiac POCUS in traumatic cardiac arrests, the frequency with which cardiac POCUS is performed has not been well studied.^30^ Although the number of patients in our center with traumatic cardiac arrest is reflective of a busy, urban Level I trauma center, more patients and more centers would help confirm the validity of our findings.

The average age in our patient population was 48 years, which is generally an older population compared to other studies. We noted 35 patients who were at least 65, each of whom presented with blunt trauma. The two most common inciting traumatic incidents in this population were motor vehicle collision and fall. Just as in our two patients who survived to hospital discharge, it is not clear whether there was a preceding medical incident that led to the traumatic injury in these patients because autopsy data was not available to us, and many patients’ families did not desire definitive autopsies. Again, to maintain consistency, we elected to enroll any patient who had a trauma activation with trauma surgery consult. Lastly, despite analyzing almost seven years’ worth of data, we found only 79 traumatic cardiac arrest cases with POCUS. We selected our study window to coincide with the implementation of our current. EHR.

## CONCLUSION

The absence of cardiac activity on ultrasound in traumatic cardiac arrest patients was associated with nonsurvivability and reduced hospital visit charges in the ED and in hospital. The ability of ED and trauma teams to ascertain the presence or absence of cardiac activity so rapidly at bedside with POCUS is an excellent reason to consider ultrasound early in resuscitation efforts. Further research, including prospective studies and those with larger sample sizes, are needed to best elucidate the best practices for cardiac point-of-care ultrasound in patients with traumatic cardiac arrest.

## Supplementary Information



## Figures and Tables

**Figure:**
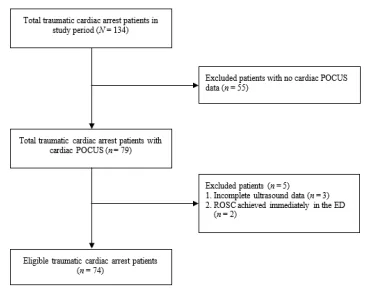
Diagram of included and excluded cases in a study of the value of point-of-care ultrasound to determine cardiac activity and outcome in traumatic cardiac arrest patients.. *ED*, emergency department; *POCUS*, point-of-care ultrasound; *ROSC*, return of spontaneous circulation.

**Table 1:** Trauma activation criteria in a study analyzing the value of point-of-care ultrasound to determine cardiac activity and outcome in patients with traumatic cardiac arrest.

GCS ≤ 12 Systolic blood pressure < 90 mm Hg Respiratory rate < 10 or > 29, intubated or with threatened airway Any penetrating injury to the head, neck, torso, or extremities proximal to the elbows or knees MVC with patient ejected from vehicle Fall > 20 feet Major deep burns (20% body surface area) Chest wall instability Two or more proximal long bone fractures Suspected unstable pelvic fracture Open or depressed skull fracture Paralysis secondary to injury Amputation proximal to wrist or ankle Crushed, degloved, or mangled extremity at or proximal to elbows or knees Pregnant patient 20 weeks or greater with vaginal bleeding, abdominal tenderness, and/or injury Transfer patients receiving blood to maintain vital signs, positive FAST examination, or chest tube insertion Emergency physician’s discretion

*FAST*, focused assessment for sonography in trauma; *GCS*, Glasgow Coma Scale; *MVC*, motor vehicle collision.

**Table 2:** Characteristics of patients with traumatic cardiac arrest who had point-of-care ultrasound performed during resuscitation efforts compared with those that did not, in a study analyzing the value of ultrasound to determine cardiac activity and outcome.

Traumatic Cardiac Arrest	POCUS Performed (*n* = 79)	No POCUS Performed (*n* = 55)	*P* value
Age	46.9 years (SD 22.3)	52.1 years (SD 22.6)	.53
Sex	61 males (77.2%)	44 males (81.8%)	.29
Blunt vs penetrating trauma	63 blunt, 16 penetrating	44 blunt, 11 penetrating	.91
Airways in ED	22 (27.8%)	18 (32.7%)	.80
Chest tube(s)	33	18	.02*
Central venous access	18	19	.88
PRBCs administered per patient (pt) in the ED	0.63/pt	1.1/pt	.06
Thoracotomies	1	4	.27
Operating room from the ED	1	5	.01*
ED death	66 (83.5%)	23 (41.8%)	.001*
If death in ED, resuscitation length prior to termination	18.44 min	29.23 min	.04*
Survival to hospital admission	12 (15.2%)	32 (58.1%)	≤ .001*
Survival to hospital discharge	3 (3.8%)	9 (16.4%)	≤ .01*
Total length of hospital stay (days)	88 (1.35/pt)	166 (4.49/pt)	≤ .01*

* Significant differences (*P* < .05) between groups.

*ED*, emergency department; *POCUS*, point-of-care ultrasound; *PRBC*, packed red blood cells; *SD*, standard deviation.

**Table 3:** Characteristics of patients with traumatic cardiac arrest who had cardiac activity as assessed on point-of-care ultrasound in resuscitation compared with those who did not, in a study analyzing the value of ultrasound to determine cardiac activity and outcome.

Traumatic Cardiac Arrest	No cardiac activity (*n* = 57)	Cardiac activity (*n* = 17)	*P* value
Age	48.5 years (SD 17.8)	47.1 years (SD 23.5)	.99
Sex	45	13	.83
Blunt vs penetrating Trauma	45 Blunt, 12 Penetrating	15 Blunt, 2 Penetrating	.39
PRBCs administered per patient (pt) in the ED	0.44/pt	0.941/pt	.01
Thoracotomies	0	1	.29
Operating room from the ED	0	1	.29
If death in ED, resuscitation length prior to termination	7.67 min	26.59 min	< .001
Survival to hospital admission	1	8	< .001
Survival to hospital discharge	0	2	.06
Total length of hospital stay (days)	1 (0.018/pt)	67 (8.38/pt)	< .001

*ED*, emergency department; *PRBC*, packed red blood cells; *SD*, standard deviation.

**Table 4:** Hospital charges based on outcomes for patients broken down by those patients that had cardiac activity on point-of-care ultrasound compared with those patients that had no cardiac activity on ultrasound, in a study of the value of ultrasound to determine cardiac activity and outcome in traumatic cardiac arrest patients.

Traumatic Cardiac Arrest	No cardiac activity (*n* = 57)	Cardiac activity (*n* = 17)	*P* value
Average charge of hospital visit	$10,032 (SD $12,976)	$91,321 (SD $133,402)	< .001
Average charge of hospital visit for patients who ultimately died in the ED	$8,945 (SD $6,501)	$27,024 (SD $19,850)	< .001
Average charge of hospital visit for patients who survived to hospital admission	$69,805	$180,991	< .001
Average charge of hospital visit for patients who survived to hospital discharge	n/a	$346,329	

*ED*, emergency department; *SD*, standard deviation.
