# Supplementary material for: Clinical and Financial Value of Cardiac Point-of-Care Ultrasound During Traumatic Cardiac Arrest
Source: West J Emerg Med. 2026 Sep 10;27(5):1280–9. doi: 10.5811/westjem.61896 (PMC13660587; doi:10.5811/westjem.61896)
Supplement: Supplementary file 1 [file wjem-27-1280-s001.docx]

Appendix

- GCS ≤ 12
- Systolic Blood Pressure < 90 mmHg
- Respiratory rate < 10 or > 29, intubated or with threatened airway
- Any penetrating injury to the head, neck, torso, or extremities proximal to the elbows or knees
- MVC with patient ejected from vehicle
- Fall > 20 feet
- Major deep burns (20% body surface area)
- Chest wall instability
- Two or more proximal long bone fractures
- Suspected unstable pelvic fracture
- Open or depressed skull fracture
- Paralysis secondary to injury
- Amputation proximal to wrist or ankle
- Crushed, degloved, or mangled extremity at or proximal to elbows or knees
- Pregnant patient 20 weeks or greater with vaginal bleeding, abdominal tenderness, and/or injury
- Transfer patients receiving blood to maintain vital signs, positive FAST examination, or chest tube insertion
- Emergency Department Physician’s discretion

Supplemental Table 1: Trauma Activation Criteria at our single institution in a study of the value of POCUS to determine cardiac activity and outcome.

| Case Number | Sex | Age | Comorbidities | Mechanism | Cardiac Activity on POCUS? | Operating Room? | Disposition |
| --- | --- | --- | --- | --- | --- | --- | --- |
| 1 | M | 59 | Hypertension, hyperlipidemia | Motor vehicle collision | Yes | No | Survival to Hospital Admission, Death in Hospital |
| 2 | M | 59 | None on File | Fall | Yes | No | Death in ED |
| 3 | F | 46 | None on File | Ski accident | Yes | No | Death in ED |
| 4 | F | 51 | None on File | Motor vehicle collision with ejection | Yes | No | Death in ED |
| 5 | M | 26 | None on File | Gunshot wound | Yes | No | Death in ED |
| 6 | M | 21 | None on File | Motor vehicle collision | Yes | No | Survival to Hospital Admission, Death in Hospital |
| 7 | M | 28 | None on File | Pedestrian struck | Yes | No | Survival to Hospital Admission, Death in Hospital |
| 8 | M | 55 | None on file | Motor vehicle collision | Yes | No | Survival to Hospital Discharge |
| 9 | M | 31 | None on file | Gunshot wound to the face | Yes | No | Survival to Hospital Admission, Death in Hospital |
| 10 | M | 27 | None on File | Motorcycle collision | Yes | No | Survival to Hospital Admission, Death in Hospital |
| 11 | M | 57 | Alcohol use disorder | Second story fall | Yes | No | Survival to Hospital Admission, Death in Hospital |
| 12 | F | 48 | None on File | Run over by dump truck | Yes | No | Death in ED |
| 13 | F | 90 | Atrial fibrillation | Fall from a stool | Yes | No | Death in ED |
| 14 | M | 60 | None on File | Motor vehicle collision | Yes | No | Death in ED |
| 15 | M | 58 | Aortic valve replacement | Motor vehicle collision | Yes | No | Death in ED |
| 16 | M | 54 | None on File | Fall off ladder | Yes | No | Survival to Hospital Discharge |
| 17 | M | 30 | None on File | Pedestrian struck | Yes | No | Death in ED |
| 18 | M | 83 | None on File | Plow strike | No | No | Death in ED |
| 19 | M | 79 | None on File | Fall down stairs | No | No | Death in ED |
| 20 | M | 81 | None on File | Pedestrian struck | No | No | Death in ED |
| 21 | M | 60 | None on File | Fall | No | No | Death in ED |
| 22 | F | 58 | None on File | Pedestrian struck | No | No | Death in ED |
| 23 | M | 37 | None on File | Motorcycle crash | No | No | Death in ED |
| 24 | F | 46 | None on File | Jump into water | No | No | Death in ED |
| 25 | M | 93 | Bronchitis, prostate cancer | Fall down stairs | No | No | Death in ED |
| 26 | M | 29 | None on File | Fall | No | No | Death in ED |
| 27 | M | 91 | Congestive heart failure, coronary artery disease | Fall | No | No | Death in ED |
| 28 | M | 23 | None on File | Fall down stairs | No | No | Death in ED |
| 29 | F | 85 | None on File |  | No | No | Death in ED |
| 30 | M | 27 | None on File | Motorcycle collision | No | No | Death in ED |
| 31 | M | 21 | None on File | Motorcycle collision | No | No | Death in ED |
| 32 | M | 46 | None on File | Motor vehicle collision | No | No | Death in ED |
| 33 | M | 52 | Osteogenesis imperfecta, stroke, hyperlipidemia, substance abuse, depression | Pedestrian struck | No | No | Death in ED |
| 34 | F | 41 | None on file | Motor vehicle collision | No | No | Death in ED |
| 35 | M | 23 | None on file | Fall down stairs | No | No | Death in ED |
| 36 | F | 48 | Depression | Large laceration | No | Yes | Survival to Hospital Admission, Death in Hospital |
| 37 | M | 34 | None on File | Gunshot wound to head and chest | No | No | Death in ED |
| 38 | F | 91 | Osteoporosis, hypertension, atrial fibrillation | Fall | No | No | Death in ED |
| 39 | M | 30 | None on File | Stab wound to chest | No | No | Death in ED |
| 40 | M | 68 | Diabetes, leukemia, coronary artery disease | Bicycle accident | No | No | Death in ED |
| 41 | M | 34 | None on File |  | No | No | Death in ED |
| 42 | F | 61 | None on file | Motor vehicle collision | No | No | Death in ED |
| 43 | M | 24 | None on file | Steel roofing collapsed on top of him | No | No | Death in ED |
| 44 | M | 57 | None on file | Found down with significant facial trauma | No | No | Death in ED |
| 45 | M | 37 | None on file | Rollover motor vehicle collision | No | No | Death in ED |
| 46 | M | n/a | None on file | Motor vehicle collision | No | No | Death in ED |
| 47 | M | 66 | None on file | Crushed by forklift | No | No | Death in ED |
| 48 | M | 60 | None on file | Struck by car | No | No | Death in ED |
| 49 | M | 33 | None on file | Bicycle accident | No | No | Death in ED |
| 50 | M | 16 | None on file | Gunshot wound to chest | No | No | Death in ED |
| 51 | M | 28 | None on file | Motorcycle accident | No | No | Death in ED |
| 52 | M | 32 | None on File | Stab wound | No | No | Death in ED |
| 53 | M | 52 | None on file | Car fell on patient | No | No | Death in ED |
| 54 | F | 21 | None on file | Motor vehicle collision | No | No | Death in ED |
| 55 | F | 19 | None on File | Gunshot wound to left and right chest | No | No | Death in ED |
| 56 | M | 49 | None on File | Rollover motor vehicle collision | No | No | Death in ED |
| 57 | M | 28 | None on File | Multiple puncture wounds in left axilla | No | No | Death in ED |
| 58 | M | 89 | Coronary artery disease, brain aneruysm, hyperlipidemia, gastroesophageal reflux disease | Fall down stairs | No | No | Death in ED |
| 59 | M | 35 | None on File | Gunshot wound to chest | No | No | Death in ED |
| 60 | M | 30 | Schizoaffective disorder, bipolar disorder | Gunshot wounds and stab wounds | No | No | Death in ED |
| 61 | M | 41 | None on File | Motor vehicle collision | No | No | Death in ED |
| 62 | M | 43 | None on File | Fall | No | No | Death in ED |
| 63 | M | 87 | None on File | Fall, ground level | No | No | Death in ED |
| 64 | M | 20 | None on File | Gunshot wound x 4 | No | No | Death in ED |
| 65 | M | 19 | None on File | Gunshot wound x 2 | No | No | Death in ED |
| 66 | M | 23 | None on File | Gunshot wound to head | No | No | Death in ED |
| 67 | F | 65 | Stroke, end-stage renal disease, seizure | Fall from 2nd floor | No | No | Death in ED |
| 68 | F | 89 | None on File | Fall down stairs | No | No | Death in ED |
| 69 | M | 53 | None on File | Fall down stairs | No | No | Death in ED |
| 70 | M | 36 | Polysubstance abuse, IV drug abuse | Found down | No | No | Death in ED |
| 71 | F | 71 | None on File | Pedestrian struck | No | No | Death in ED |
| 72 | M | 76 | None on File | Motor vehicle collision | No | No | Death in ED |
| 73 | M | 30 | None on File | Motorcycle accident | No | No | Death in ED |
| 74 | M | n/a | None on File | Gunshot wound x 11 | No | No | Death in ED |

Supplemental Table 2: Patient breakdown with their age, sex, comorbidities, presence of cardiac activity and their disposition, in a study of the value of POCUS to determine cardiac activity and outcome in traumatic cardiac arrest.

| Case Number | Race | Ethnicity | Airway | Chest tube | Central Access | Thoracotomy | Blood Products  (units of PRBCs) |
| --- | --- | --- | --- | --- | --- | --- | --- |
| 1 | White or Caucasian | Not Hispanic or Latino | Initial Intubation Prehospital, Re-Intubated in ED | None | No | No | 0 |
| 2 | White or Caucasian | Not Hispanic or Latino | Prehospital Intubation | Unilateral | No | No | 2 |
| 3 | White or Caucasian | Not Hispanic or Latino | Prehospital Intubation | Bilateral | No | No | 0 |
| 4 | White or Caucasian | Not Hispanic or Latino | ED Intubation | None | Yes | No | 1 |
| 5 | Black or African American | Not Hispanic or Latino | Prehospital Intubation | None | No | No | 0 |
| 6 | Black or African American | Not Hispanic or Latino | Prehospital Intubation | Bilateral | Yes | No | 0 |
| 7 | White or Caucasian | Not Hispanic or Latino | Prehospital Intubation | None | No | No | 2 |
| 8 | White or Caucasian | Not Hispanic or Latino | Prehospital Intubation | None | Yes | No | 0 |
| 9 | Black or African American | Not Hispanic or Latino | Prehospital Intubation | None | Yes | No | 4 |
| 10 | Black or African American | Not Hispanic or Latino | ED Intubation | Unilateral | No | No | 0 |
| 11 | Other | Hispanic or Latino | ED Intubation | None | Yes | No | 4 |
| 12 | White or Caucasian | Not Hispanic or Latino | Prehospital Intubation | None | No | No | 0 |
| 13 | White or Caucasian | Not Hispanic or Latino | ED Intubation | None | No | No | 0 |
| 14 | Other | Hispanic or Latino | Failed, LMA | None | No | No | 2 |
| 15 | White or Caucasian | Not Hispanic or Latino | ED Intubation | None | No | No | 0 |
| 16 | White or Caucasian | Not Hispanic or Latino | Prehospital Intubation | Bilateral | No | No | 0 |
| 17 | Unknown | Unknown | Prehospital Intubation | Bilateral | Yes | No | 1 |
| 18 | White or Caucasian | Hispanic or Latino | Prehospital Intubation | Bilateral | No | No | 0 |
| 19 | White or Caucasian | Not Hispanic or Latino | Prehospital Intubation | None | No | No | 0 |
| 20 | White or Caucasian | Not Hispanic or Latino | Prehospital Intubation | None | No | No | 0 |
| 21 | White or Caucasian | Not Hispanic or Latino | Prehospital Intubation | Unilateral | No | No | 0 |
| 22 | Black or African American | Not Hispanic or Latino | ED Intubation | Unilateral | Yes | No | 1 |
| 23 | White or Caucasian | Not Hispanic or Latino | Prehospital Intubation | Bilateral | No | No | 0 |
| 24 | White or Caucasian | Not Hispanic or Latino | Initial Intubation Prehospital, Re-Intubated in ED | Unilateral | No | No | 0 |
| 25 | White or Caucasian | Not Hispanic or Latino | Prehospital Intubation | None | No | No | 0 |
| 26 | Black or African American | Not Hispanic or Latino | Prehospital Intubation | None | No | No | 0 |
| 27 | White or Caucasian | Not Hispanic or Latino | Prehospital Intubation | None | No | No | 0 |
| 28 | White or Caucasian | Not Hispanic or Latino | Prehospital Intubation | Unilateral | No | No | 0 |
| 29 | Other | Hispanic or Latino | Prehospital Intubation | None | No | No | 0 |
| 30 | Other | Hispanic or Latino | BVM | None | No | No | 0 |
| 31 | White or Caucasian | Not Hispanic or Latino | Prehospital Intubation | None | No | No | 0 |
| 32 | Unknown | Unknown | Prehospital Intubation | Bilateral | No | No | 0 |
| 33 | Other | Hispanic or Latino | Cricothyrotomy | None | No | No | 0 |
| 34 | White or Caucasian | Not Hispanic or Latino | Prehospital Intubation | None | No | No | 0 |
| 35 | White or Caucasian | Not Hispanic or Latino | Prehospital Intubation | None | Yes | No | 0 |
| 36 | White or Caucasian | Not Hispanic or Latino | Prehospital Intubation | None | Yes | No | 9 |
| 37 | Other | Hispanic or Latino | Prehospital Intubation | Bilateral | No | Yes | 6 |
| 38 | White or Caucasian | Not Hispanic or Latino | Prehospital Intubation (cric!) | None | No | No | 0 |
| 39 | Black or African American | Not Hispanic or Latino | BVM | None | No | No | 0 |
| 40 | White or Caucasian | Not Hispanic or Latino | ED Intubation | None | No | No | 0 |
| 41 | Unknown | Unknown | ED Intubation | Bilateral | Yes | No | 1 |
| 42 | White or Caucasian | Not Hispanic or Latino | Prehospital Intubation | Unilateral | No | No | 1 |
| 43 | Other | Hispanic or Latino | Prehospital Intubation | None | No | No | 0 |
| 44 | Unknown | Unknown | Initial Intubation Prehospital, Re-Intubated in ED | None | Yes | No | 0 |
| 45 | Other | Hispanic or Latino | ED Intubation | None | No | No | 0 |
| 46 | Unknown | Unknown | Prehospital Intubation | None | No | No | 0 |
| 47 | White or Caucasian | Not Hispanic or Latino | Prehospital Intubation | None | No | No | 0 |
| 48 | Black or African American | Not Hispanic or Latino | ED Intubation | None | No | No | 0 |
| 49 | Black or African American | Not Hispanic or Latino | Prehospital Intubation | None | No | No | 0 |
| 50 | Black or African American | Hispanic or Latino | Prehospital Intubation | Bilateral | No | No | 0 |
| 51 | Other | Hispanic or Latino | ED Intubation | Bilateral | No | No | 0 |
| 52 | Other | Hispanic or Latino | ED Intubation | None | No | No | 2 |
| 53 | Unknown | Unknown | BVM | None | No | No | 0 |
| 54 | Unknown | Unknown | Prehospital Intubation | None | No | No | 0 |
| 55 | Unknown | Unknown | Supraglottic tube | None | No | No | 0 |
| 56 | White or Caucasian | Not Hispanic or Latino | Prehospital Intubation | None | No | No | 0 |
| 57 | Black or African American | Not Hispanic or Latino | Supraglottic tube | Bilateral | No | No | 0 |
| 58 | Other | Hispanic or Latino | Bagged | None | No | No | 0 |
| 59 | Other | Hispanic or Latino | Initial Intubation Prehospital, Re-Intubated in ED | None | No | No | 0 |
| 60 | Black or African American | Hispanic or Latino | ED Intubation | None | No | No | 0 |
| 61 | White or Caucasian | Not Hispanic or Latino | Prehospital Intubation | Bilateral | Yes | No | 0 |
| 62 | Black or African American | Not Hispanic or Latino | ED Intubation | None | No | No | 0 |
| 63 | Black or African American | Not Hispanic or Latino | BVM | None | No | No | 0 |
| 64 | Other | Hispanic or Latino | Prehospital Intubation | None | No | No | 0 |
| 65 | Other | Hispanic or Latino | ED Intubation | None | No | No | 0 |
| 66 | Black or African American | Not Hispanic or Latino | ED Intubation | None | No | No | 0 |
| 67 | Other | Hispanic or Latino | ED Intubation | Bilateral | Yes | No | 0 |
| 68 | Unknown | Unknown | ED Intubation | Bilateral | Yes | No | 0 |
| 69 | Other | Not Hispanic or Latino | ED Intubation | None | No | No | 0 |
| 70 | White or Caucasian | Not Hispanic or Latino | ED Intubation | None | No | No | 0 |
| 71 | Black or African American | Not Hispanic or Latino | ED Intubation | None | No | No | 0 |
| 72 | White or Caucasian | Not Hispanic or Latino | ED Intubation | Bilateral | Yes | No | 2 |
| 73 | Other | Hispanic or Latino | Prehospital Intubation | None | Yes | No | 3 |
| 74 | Unknown | Unknown | ED Intubation | Bilateral | No | No | 0 |

Supplemental Table 3: Additional demographics and procedures conducted for patients, in a study of the value of POCUS to determine cardiac activity and outcome in traumatic cardiac arrest.
